# Intravenous tacrolimus is a superior induction therapy for acute severe ulcerative colitis compared to oral tacrolimus

**DOI:** 10.1186/s12876-021-02043-6

**Published:** 2021-12-23

**Authors:** Hiromichi Shimizu, Toshimitsu Fujii, Kenji Kinoshita, Ami Kawamoto, Shuji Hibiya, Kento Takenaka, Eiko Saito, Masakazu Nagahori, Kazuo Ohtsuka, Mamoru Watanabe, Ryuichi Okamoto

**Affiliations:** 1 Department of Gastroenterology and Hepatology, Tokyo Medical and Dental University, 1-5-45 Yushima, Bunkyo-ku, Tokyo, 113-8519 Japan; 2 Department of Endoscopy, Tokyo Medical and Dental University, Tokyo, Japan; 3 Department of Gastroenterology, Hakodate Hospital, Hakodate, Hokkaido Japan

**Keywords:** Ulcerative colitis, Acute severe ulcerative colitis, Intravenous tacrolimus

## Abstract

**Background:**

Intravenous corticosteroid is the mainstay for managing acute severe ulcerative colitis, but one-third of patients do not respond to intravenous corticosteroid. Tacrolimus, a salvage therapy before colectomy, is usually orally administered, though its bioavailability is low compared intravenous administration. The efficacy of intravenous tacrolimus has not been widely studied.

**Aim:**

To determine the efficacy and safety of intravenous tacrolimus for the treatment of acute severe ulcerative colitis.

**Methods:**

Eighty-seven hospitalized acute severe ulcerative colitis patients were enrolled for a prospective cohort study between 2009 and 2017. Sixty-five patients received intravenous tacrolimus and 22 received oral tacrolimus. The primary outcome was the achievement of clinical remission within 2 weeks. Relapse and colectomy incidence and adverse events were assessed at 24 weeks.

**Results:**

Response rates of both treatments exceeded 50% but were not significantly different. The remission rate was higher in intravenous tacrolimus compared with oral tacrolimus. At 24 weeks, oral and intravenous tacrolimus showed similar relapse-free survival rates; however, colectomy-free survival rates were higher in intravenous tacrolimus compared with oral tacrolimus.

**Conclusions:**

Patients receiving intravenous tacrolimus achieved superior remission and colectomy-free survival rates compared with patients receiving oral tacrolimus. Safety was similar between the two treatments.

## Introduction

Ulcerative colitis (UC) is an inflammatory bowel disease characterized by chronic inflammation of the colon. More than 20% of patients with UC will develop acute severe ulcerative colitis (ASUC) and of those patients, up to 30% fail to adequately respond to high-dose intravenous corticosteroid [1]. If corticosteroid treatment does not improve the disease immediately, the patients are at risk of mortality and colectomy is the only available treatment option [2–4]. Recent advances, including the development of calcineurin inhibitors (CNIs), cyclosporine, tacrolimus and a novel anti-tumor necrosis factor (TNF)-α antibody, infliximab, provide alternative treatment options before colectomy. Salvage therapies with these medications have greatly improved the prognosis of ASUC patients. CNIs or infliximab are the best options after failure of high-dose intravenous corticosteroid [5–8]. The use of CNIs versus infliximab for induction of salvage therapy is controversial. Infliximab can be continued as maintenance therapy after induction. However, the increasing use of infliximab or other anti-TNF-α antibodies can lead the patients to fail anti-TNF-α therapies or become intolerant due to adverse events. Thus, CNIs can still be an alternative option for ASUC patients to escape acute severe disease and emergent colectomies can serve as a bridging therapy for maintenance.

Tacrolimus (TAC), one of the CNIs, is an immunosuppressive macrolide isolated from Streptomyces tsukubaensis. TAC, which is structurally different from cyclosporin, selectively binds to FK-binding protein 12 to inhibit calcineurin and reduce transcription of interleukin (IL)-2, IL-4, IL-10, IL-17 and interferon-γ [9, 10]. Decreased cytokine transcription eventually suppresses the inflammatory response. TAC has two formulations, an oral capsule and intravenous injection. For the treatment of UC, oral TAC is generally used and approved in our country. When orally administered, the bioavailability of TAC is relatively low and unstable. Oral TAC reaches an effective trough concentration in 3–5 days, which is too long for patients with ASUC. Oral TAC reaches a peak concentration 1–3 h after administration and has a 12-h half-life, which means it has multimodal pharmacodynamics during the day [11].

We hypothesized that the efficacy of TAC toward ASUC could be improved with intravenous administration which is used as continuous infusion. Intravenous TAC reaches effective concentrations faster and the best concentration can be stably reached at any time of the day. Here, we showed the results analyzing the efficacy of intravenous TAC for corticosteroid refractory ASUC patients.

## Materials and methods

We performed a prospective cohort study investigating the efficacy of intravenous TAC between April 2009 and September 2017, at the Tokyo Medical and Dental University Hospital. ASUC patients of 15 years or older were included in the study, and patients with known kidney disorder or cancer history and pregnant women were excluded from the study. A total of 87 hospitalized ASUC patients were enrolled in this study, among which 65 patients accepted to receive intravenous TAC. Twenty-two patients did not accept it and received oral TAC. ASUC was defined by a Lichtiger index (LI) of more than 10 points [12]. We collected data on demographics, disease duration, disease types, calculated clinical activity using the LI, treatment histories, laboratory tests and endoscopic scores (ulcerative colitis endoscopic index of severity; UCEIS) [13] when the patients started TAC. All patients received unsuccessful courses of high-dose intravenous corticosteroid therapy (0.5–1.0 mg/kg body weight per day of prednisolone) before TAC therapy.

TAC was administered as described below. Whole blood concentrations of tacrolimus were monitored daily during hospitalization; the dose target was set between the effective concentration of 10 ng/ml and toxic concentration of 20 ng/ml according to the manufacturer’s instructions. The dose was adjusted according to the concentration. The treatment protocol of intravenous TAC administration was decided in accordance with that of bone marrow transplantation. Patients receiving intravenous TAC were started at 0.025 mg/kg body weight per day as a continuous intravenous infusion and adjusted to a target concentration of 15–20 ng/ml. Seven days after starting intravenous TAC, patients were switched to oral TAC at 0.10–0.15 mg/kg body weight per day. Oral TAC was continued to keep the trough concentration at 10–15 ng/ml for another 7 days. For patients receiving only oral TAC, the administration started at 0.1 mg/kg body weight per day [14] and the dose was adjusted to target trough concentration of 10–15 ng/ml for 2 weeks [15]. After successful induction therapy, the trough concentration was set between 5 and 10 ng/ml and TAC was continued as long as it was effective without any adverse events for 3 months or longer, combined with an immunomodulator for maintenance therapy.

The primary outcome was the achievement of clinical remission within 2 weeks, defined by the LI. Patients were evaluated daily through self-written symptom diary, recording LI and adverse events that disturbed the treatment leading to dose reduction or discontinuation. For long-term outcomes, relapse and colectomy incidence and adverse events were assessed for 24 weeks. The outpatient follow-up was not pre-defined but decided by the attending physicians. We defined remission as LI ≤ 4 points and response as LI < 10 points with a decrease of at least 3 points. Treatment failure was defined as TAC discontinuation due to nonresponse to the medication, symptom recurrence in patients, and/or serious adverse events. Lastly, relapse was defined as TAC intensification or discontinuation due to symptom recurrence in patients who had already achieved remission.

For statistical analyses, differences in the medians between groups were compared using non-parametric tests (Mann–Whitney’s test) and comparisons between categorical variables were analyzed using Fisher’s exact tests. We calculated relapse-free survival and colectomy-free survival and generated Kaplan–Meier curves for each treatment group, then compared them with log-rank (Mantel–Cox) tests. For further analyses, we used univariate and multivariable logistic regression models to determine predictors of colectomy. Multivariable logistic regression analysis was performed using the following variables known as risk factors for colectomy: male sex, extensive disease type, LI, endoscopic score (UCEIS), and C-reactive protein [13]. We used SPSS (version 26.0.0.0) software for our statistical analysis, and the significance threshold was 0.05 for every analysis.

This study was performed in accordance with relevant guidelines and regulations, and the Tokyo Medical and Dental University Institutional Review Board approved the study (IRB No. 20101104). All patients provided written informed consent and for patients under 18 years, patient’s parent and/or legal guardian provided written informed consent. All data analyzed in this study are available from the corresponding author on reasonable request.

## Results

### Patient characteristics

Between April 2009 and September 2017, 128 UC patients were hospitalized in our facility and received TAC for the treatment of corticosteroid refractory and/or dependent disease. Of those patients, 87 patients were diagnosed with ASUC, including 65 patients receiving intravenous TAC and 22 patients receiving oral TAC. Patient characteristics at the time they started TAC therapy are shown in Table 1. Thirty-nine female and 48 male patients were included in the study and the median age was 40 years (range 24–50) when TAC therapy was started. The median disease duration was 2.9 years (range 1.0–8.3). UC was extensive in 71 patients (82%) and left-sided in 16 patients (18%). Sixty-six patients (76%) and 21 patients (24%) were corticosteroid refractory and dependent, respectively. Sixty-one patients (70%) were biologic agent naïve. The median LI was 13 (range 12–15) and the mean endoscopic score (UCEIS) was 5.9 ± 1.0. All characteristics were statistically similar between the oral and intravenous TAC groups.

**Table 1:** Patient characteristics when tacrolimus treatment was started

	Overall (N = 87)	Oral (N = 22)	Intravenous (N = 65)	*p* value
Female/male	39 (45%)/48 (55%)	8 (36%)/14 (64%)	31 (48%)/34 (52%)	0.46
Age (years)	40 (24–50)	39 (25–49)	40 (24–52)	0.57
Disease duration (years)	2.9 (1.0–8.3)	2.5 (0.8–11.5)	3.0 (1.2–7.8)	0.95
Disease type: extensive /left-sided	71 (82%)/16 (18%)	16 (73%)/6 (27%)	55 (85%)/10 (15%)	0.18
Body mass index	19.4 (16.9–22.6)	20.1 (16.6–23.6)	19.4 (16.8–22.1)	0.8
Lichtiger index	13 (12–15)	12 (12–14)	14 (12–15)	0.09
Endoscopic score (UCEIS)	6 (5–7)	6 (4–8)	6 (5–7)	0.86
Hemoglobin (g/dL)	11.3 (9.7–12.8)	12.0 (10.3–13.4)	11.3 (9.2–12.8)	0.13
Albumin (g/dL)	3.2 (2.6–3.7)	3.2 (2.6–3.8)	3.2 (2.6–3.7)	0.74
C-reactive protein (mg/dL)	3.1 (1.5–9.0)	4.2 (2.2–11.8)	2.8 (1.1–7.4)	0.06
Erythrocyte sedimentation rate (mm/h)	53 (32–81)	56 (31–73)	48 (35–82)	0.87
Creatinine (mg/dL)	0.7 (0.6–0.8)	0.7 (0.6–0.8)	0.7 (0.6–0.8)	0.83
Corticosteroid refractory/dependent	66 (76%) / 21 (24%)	16 (73%) / 6 (27%)	50 (77%) / 15 (23%)	0.45
Biologic agent naive	61 (70%)	14 (64%)	47 (72%)	0.31

Numerical data shows median value with interquartile range. Continuous variables were compared using the Mann–Whitney U test and categorical variables were compared using Fischer’s exact test

*UCEIS* ulcerative colitis endoscopic index of severity

### Tacrolimus concentration

**Fig. 1:**
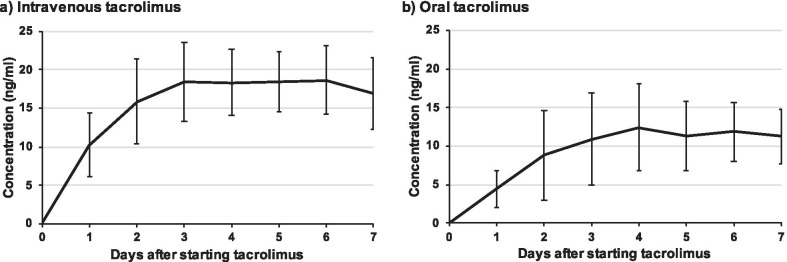
Tacrolimus concentrations. Tacrolimus concentration curves showed rapid achievement of effective concentrations for intravenous tacrolimus. Intravenous tacrolimus reached effective concentrations 24 h after first administration, whereas oral administration reached effective concentrations at day 3 on average. After reaching effective concentrations, intravenous tacrolimus plateaued at its target concentration from day 2

The daily whole blood concentrations of TAC are shown in Fig. 1. Intravenous administration elevated the mean concentration to 10.2 ± 4.2 ng/ml within 24 h after the first administration. On the other hand, the trough concentration in the oral administration group at the same time point was 4.4 ± 2.4 ng/ml, and effective trough concentration was achieved on day 3 on average. After achieving effective concentration, intravenous TAC plateaued at the target concentration after day 2. The dose of TAC was continually adjusted within the target ranges in accordance with the protocol.

### Efficacy of tacrolimus

**Fig. 2:**
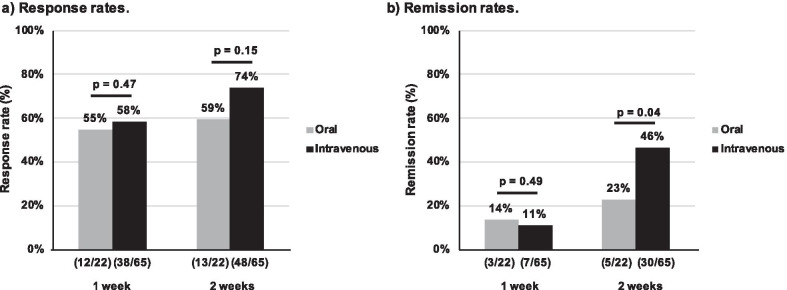
Efficacy of tacrolimus. **a** Response rates. Response rates of both tacrolimus groups exceeded 50% at 1 week with no significant differences were detected at 1 or 2 weeks. **b** Remission rates. Remission rates at 2 weeks after oral and intravenous tacrolimus administration were 23% and 46%, respectively. Intravenous tacrolimus was significantly superior to oral administration at 2 weeks (*p* = 0.04)

**Fig. 3:**
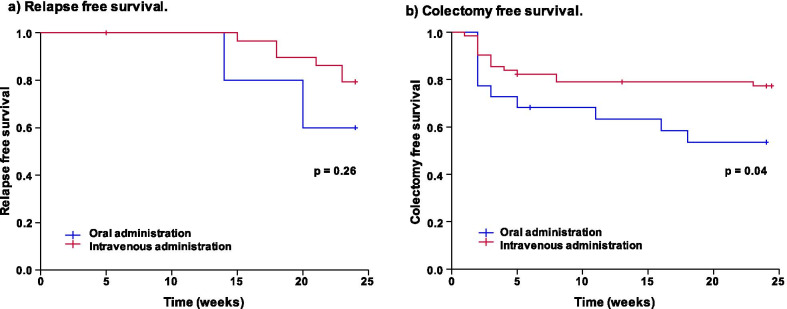
Twenty-four weeks efficacy of intravenous tacrolimus. **a** Relapse-free survival at 24 weeks. Among the patients who achieved remission at 2 weeks, oral and intravenous tacrolimus groups showed similar 24-week relapse-free survival rates of 60.0% ± 21.9% and 79.3% ± 7.5%, respectively. **b** Colectomy-free survival at 24 weeks. The 24-week colectomy-free survival rates in patients treated with intravenous tacrolimus (76.8% ± 5.3%) were better than the survival rates in patients treated with oral TAC (53.6% ± 10.8%)

Short-term efficacy at 1 week and 2 weeks of TAC administration are shown in Fig. 2. One-week response rates for oral and intravenous TAC were 55% and 58%, respectively. The two-week response rates were 59% and 74%, respectively. No significant differences in one- and two-week response rates were detected (Fig. 2a). One-week remission rates for oral and intravenous TAC were similar (14% and 11%, respectively, *p* > 0.05). However, the 2-week remission rate for intravenous TAC (46%) was significantly higher than the remission rate for oral TAC (23%) (*p* = 0.04) (Fig. 2b). Descriptive statistics for remission and non-remission patients at 2 weeks showed that lower endoscopic scores and intravenous administration were associated with remission at 2 weeks. A decrease of LI ≥ 3 points by day 3 and response at 1 week were also related to the outcome (Table 2). Analyzing 24-week efficacies among patients who achieved remission at 2 weeks showed that oral and intravenous groups had similar relapse-free survival rates (60.0% ± 21.9% vs. 79.3% ± 7.5%) (Fig. 3a). However, patients treated with intravenous TAC had significantly higher colectomy-free survival rates (76.8% ± 5.3%) than patients treated with oral TAC (53.6% ± 10.8%) (*p* = 0.04) (Fig. 3b). Patients who achieved remission with oral and intravenous TAC started immunomodulators a half week (0–1 week) and a week (0.5–1.5 weeks) after starting TAC (*p* = 0.59), respectively. Those patients continued TAC for 34 weeks (0–79) and 26.5 weeks (0–54) (*p* = 0.93), respectively. For long-term efficacy, intravenous TAC was not superior to oral TAC in relapse-free survival and colectomy-free survival at 48 weeks. We performed univariable and multivariable analyses using the Cox proportional hazards model. Univariable analysis showed that higher endoscopic scores (UCEIS ≥ 6) were associated with a significantly high hazard ratio of 4.60 [95% CI 1.37–15.46] (*p* = 0.01) for colectomy at 24 weeks. A multivariable analysis demonstrated that higher endoscopic score (UCEIS ≥ 6) and oral TAC administration were associated with significantly high hazard ratios for colectomy at 24 weeks (4.39 [95% CI 1.40–13.78], *p* = 0.01 and 4.13 [95% CI 1.61–10.57], *p* < 0.01, respectively) (Table 3).

**Table 2:** Comparison of patient characteristics between remission and non-remission patients after 2 weeks of tacrolimus administration

Whole tacrolimus	Remission at 2 weeks	Non-remission at 2 weeks	*p* value
(N = 87)	(N = 35)	(N = 52)	
Female/male	16 (46%)/19 (54%)	23 (44%)/29 (56%)	0.53
Age (years)	36 (23–54)	42 (28–50)	0.42
Disease duration (years)	4.0 (1.0–9.2)	2.7 (1.1–7.0)	0.75
Disease type: extensive/left-sided	31 (89%)/4 (11%)	40 (77%)/12 (23%)	0.14
Body mass index	19.4 (16.4–22.5)	19.6 (17.0–22.2)	0.77
Lichtiger index	13 (12–14)	13 (12–15)	0.81
Endoscopic score (UCEIS)	6 (5–7)	6 (4–8)	0.03
Hemoglobin (g/dL)	10.3 (10.0–12.7)	11.8 (9.5–13.1)	0.44
Albumin (g/dL)	3.2 (2.7–3.7)	3.3 (2.6–3.7)	0.85
C-reactive protein (mg/dL)	3.8 (0.9–10.2)	3.1 (1.6–7.6)	0.96
Erythrocyte sedimentation rate (mm/h)	48 (30–83)	54 (33–75)	0.93
Creatinine (mg/dL)	0.7 (0.6–0.8)	0.7 (0.6–0.8)	0.92
Corticosteroid refractory/dependent	23 (66%)/12 (34%)	43 (83%)/9 (17%)	0.06
Biologic agent naive	28 (80%)	33 (64%)	0.08
Intravenous tacrolimus	30 (86%)	35 (67%)	0.04
Decrease of Lichtiger index ≥ 3 points by day 3	21 (60%)	11 (21%)	< 0.01
response at 1 week (LI decrease by 3 and less than 10)	32 (91%)	18 (35%)	< 0.01
Tacrolimus concentration ≥ 10 ng/ml at day 1	21 (60%)	26 (50%)	0.24
Tacrolimus concentration ≥ 10 ng/ml at day 3	31 (89%)	42 (81%)	0.25
Adverse events	10 (29%)	8 (15%)	0.11

Numerical data shows median value with interquartile range. Continuous variables were compared using the Mann–Whitney U test and categorical variables were compared using Fischer’s exact test

*UCEIS* ulcerative colitis endoscopic index of severity

**Table 3:** Cox proportional hazards model showing hazard ratios for colectomy (N = 87)

Variables at admission	Case number	Crude		Multivariable	
		Hazard ratio [95% CI]	*p* value	Hazard ratio [95% CI]	*p* value
Sex					
Female	37				
Male	47	1.34 [0.59–3.06]	0.49	1.22 [0.51–2.97]	0.66
Age					
< 39	40				
≥ 39	44	0.94 [0.42–2.09]	0.88		
Disease duration (years)					
< 2.6	40				
≥ 2.6	44	0.50 [0.22–1.15]	0.1		
Disease type					
Extensive	69	1.13 [0.39–3.31]	0.82	0.81 [0.29–2.27]	0.69
Left	15				
Lichtiger index					
< 13	29				
≥ 13	55	1.40 [0.58–3.37]	0.46	2.20 [0.80–6.08]	0.13
Endoscopic score (UCEIS)					
< 6	30				
≥ 6	54	4.60 [1.37–15.46]	0.01	4.39 [1.40–13.78]	0.01
Hemoglobin (g/dL)					
≤ 11.0	37	1.15 [0.51–2.56]	0.74		
> 11.0	47				
Albumin (g/dL)					
≤ 3.1	41	1.60 [0.71–3.59]	0.26		
> 3.1	43				
C-reactive protein (mg/dL)					
< 3.4	42				
≥ 3.4	42	0.85 [0.38–1.90]	0.7	0.47 [0.19–1.13]	0.92
Erythrocytes sedimentation rates (mm)					
< 53	41				
≥ 53	43	2.20 [0.94–5.14]	0.07		
Corticosteroid dependent					
Yes	19	0.66 [0.23–1.93]	0.45		
No	65				
Biologic agent failure					
Yes	23	2.18 [0.97–4.92]	0.06		
No	61				
Oral tacrolimus administration					
Yes	22	2.24 [0.99–5.05]	0.05	4.13 [1.61–10.57]	< 0.01
No	62				

Univariable analyses showed that higher endoscopic scores (ulcerative colitis endoscopic index of severity ≥ 6) were associated with a significantly higher hazard ratio of 4.60 [95% CI 1.37–15.46] (*p* = 0.01) for colectomy at 24 weeks. Multivariable analysis showed that higher endoscopic scores (ulcerative colitis endoscopic index of severity ≥ 6) and oral tacrolimus administration were associated with significantly higher hazard ratios for colectomy at 24 weeks of 3.98 [95% CI 1.11–14.28] (*p* = 0.03) and 3.35 [95% CI 1.17–9.56] (*p* = 0.02), respectively

*CI* confidence interval

*UCEIS* ulcerative colitis endoscopic index of severity

### Safety of intravenous tacrolimus

Twenty-seven percent of patients treated with oral TAC and 31% of patients treated with intravenous TAC experienced adverse events during the study (*p* > 0.05). Most adverse events were non-serious, including tremors, nausea, hot flashes, and peripheral neuropathies. Those adverse events did not require TAC discontinuation or any additional treatment. Kidney disorder was a common adverse event found in 4 patients; these patients had slight kidney disorders and recovered after TAC dose reduction but not discontinuation. However, one patient in the intravenous TAC group had pneumonia, which required discontinuation of TAC and colectomy. There were no significant differences in the incidence of each adverse event between the two groups (Table 4).

**Table 4:** Safety of intravenous tacrolimus

	Oral (N = 22)	Intravenous (N = 65)	*p* value
Whole adverse events	8 (36%)	20 (31%)	0.79
Tremor	4 (18%)	8 (12%)	0.49
Nausea	1 (5%)	3 (5%)	1
Hot flush	1 (5%)	2 (3%)	1
Kidney disorder	2 (9%)	2 (3%)	0.26
Peripheral neuropathy	1 (5%)	2 (3%)	1
Headache		1 (2%)	
Tachycardia		1 (2%)	
Rash		1 (2%)	
Hematopenia		1 (2%)	
Pneumonia		1 (2%)	

Less than one-third of patients experienced adverse events in both tacrolimus groups during the study. Most adverse events were non-serious, including tremors, nausea, hot flashes, and peripheral neuropathies, and did not require tacrolimus discontinuation or additional treatment. Kidney disorder was a common adverse event; four patients had slight kidney disorders that recovered with dose reduction of tacrolimus and did not require tacrolimus discontinuation. One patient had pneumonia which required discontinuation of tacrolimus and colectomy

Categorical variables were compared using Fischer’s exact test

## Discussion

Current advances have led to innovative medications, including two new anti-TNF-α antibodies, an anti-α4β7 antibody, an anti-IL12/23 antibody, and a Janus kinase inhibitor, for moderate to severe UC patients. However, we have limited practical experience and knowledge in using these medications in patients with ASUC who are at risk for colectomy; the therapeutic strategy for ASUC has not changed yet. The development of new therapeutic strategies to achieve better prognoses in ASUC patients is needed. Intravenous corticosteroid therapy is the mainstay of management for ASUC. If corticosteroid does not improve the disease in 3 to 5 days, either CNIs or infliximab therapies are tried before the patient progresses to colectomy. Salvage therapies with these medications improve the disease quickly and decrease the risk of colectomy in short time periods [7, 8]. Several reports demonstrated that CNIs and infliximab have similar long-term efficacies in terms of colectomy-free survival for years, although more patients initially treated with CNIs discontinued the medication and needed a new one [14, 16–20]. While CNIs are effective for induction, prolonged use of CNIs may cause adverse events. Patients who achieve remission with CNIs are usually transitioned to immunomodulators for maintenance. Thus, CNIs are an induction therapy or a bridging therapy, and not a long-term or maintenance therapy [21].

Here, we focused on the short-term use of TAC and described the efficacy and safety profile of intravenous TAC for ASUC. Patients treated with intravenous TAC reached an effective concentration faster than patients treated with oral TAC. Furthermore, a stable concentration of TAC was easier to attain and more sustainable when using intravenous TAC. Intravenous TAC reached the effective concentration within 24 h after the first administration and the concentration quickly plateaued to the target concentration. In contrast, oral TAC only reached the effective trough concentration on day 3 and plateaued later. The response rates of both oral and intravenous TAC were excellent but without significant differences, at 1 week (55% and 58%) and 2 weeks (59% and 74%, respectively). The fast responses were preferable; the treatment of ASUC requires fast improvement before patients need colectomies. When it comes to remission induction, the rates of oral and intravenous TAC were 14% and 11% at 1 week and 23% and 46% at 2 weeks, respectively. The remission rates in patients treated with intravenous TAC were significantly higher than the rates in patients treated with oral TAC at 2 weeks.

A decrease in the LI on day 3 enabled us to examine whether TAC was effective in the urgent situation. The response rate at 1 week was a predictive factor for 2-week remission. Also, we showed that the colectomy-free survival rates of oral and intravenous TAC were 53.6% and 76.8% at 24 weeks, and Cox proportional hazards model showed oral TAC administration was a distinct risk factor for colectomy during this same period. We assumed that both rapid and continuous achievement of effective concentrations during the acute flare of colonic inflammation contributed to the superior efficacy of intravenous TAC, even when intravenous TAC was used for only the first 1 week before switching to oral TAC. Moreover, intravenous TAC was better than oral TAC in regards to economic effect; total dose was approximately 10 mg vs. 60 mg/ 60 kg body weight for the first week, respectively.

TAC requires therapeutic drug monitoring due to its highly variable pharmacokinetics and narrow therapeutic window. Orally administered TAC would have to get absorbed first, then it circulates in the blood flow and distributes in the tissue. Simultaneously, part of it would start to get eliminated. When the rate of administration equals the rate of elimination, the drug concentration reaches steady state. Depending on the doses of oral TAC repeatedly administered twice a day, its drug concentration has peaks and troughs. Because of this peak and trough drug kinetics, it is difficult to elevate target trough concentration higher without any adverse effects that are dependent on TAC concentration. We presume oral TAC concentration by measuring trough concentrations and adjust it high enough within the effective concentration but lower than toxic level.

On the other hand, intravenously administered TAC would directly enter into the blood circulation and distribute in the tissue. Intravenous TAC is administered as continuous infusion, its concentration has no peak and trough but continuously stable. We can measure its direct concentration and adjust it directly. The use of intravenous TAC allowed tighter control at higher concentrations very stably without reaching toxic doses, which could cause adverse effects. The average bioavailability of orally administered TAC is only 25% but ranges from 5 to 90% [22]. Cytochrome P450 3A is the metabolizing enzyme for TAC. Cytochrome P450 is expressed in the small intestine and liver. While a majority of TAC is metabolized in the liver, a considerable amount of TAC is pre-systemically bio-transformed in the small intestine. Thus, the first-pass effect through the small intestine and liver contributes to the poor bioavailability of TAC. Age, body weight, drug interactions, and genetic background of cytochrome P450 3A polymorphisms contribute to the interpatient variability of TAC metabolism, making the achievement of an optimal dose of orally administered TAC more difficult in individual patients [23]. Intravenous TAC is a way to circumvent the low bioavailability of oral TAC and allows the target concentration to be reached rapidly, continuously, and stably. This superior aspect very much contributed to its safety profile; we found no differences in adverse events between oral and intravenous TAC. Major adverse events, such as nephrotoxicity, hypertension, tremor, peripheral neuropathy and headache, are dependent on TAC concentration [21]. The use of intravenous TAC allowed tighter control of the concentrations so higher concentrations could be achieved without reaching toxic doses, which could cause adverse events. As a result, more ASUC patients achieved remission at 2 weeks and better survival from colectomy at 24 weeks via intravenous TAC, which was as safe as oral TAC.

Regarding long-term efficacy, we studied our cohort for a year and did not find any significant differences between oral and intravenous TAC either in relapse-free survival or colectomy-free survival. Infliximab has a great advantage over CNIs for long-term use, as infliximab can be continued for maintenance after induction. Infliximab is also easier to administer than CNIs, as it does not require adjusting concentration. In addition, fewer adverse events occurred with infliximab use. As a result, the use of infliximab is increasing. Increasing use of infliximab or other anti-TNF-α antibodies is contributing to the increasing number of patients who fail anti-TNF-α therapies. As an increasing number of ASUC patients no longer respond to anti-TNF-α therapies, CNIs can serve as a bridging therapy to escape both acute severe disease and emergent colectomy. Recent reports showed the efficacy of sequential treatment for UC, starting with TAC for induction followed by vedolizumab for maintenance [24, 25]. The combination of TAC and vedolizumab is potentially a breakthrough idea providing an alternative therapy for ASUC patients who are anti-TNF-α naïve or no longer respond to anti-TNF antibodies. Because vedolizumab has an excellent safety profile and long-term efficacy with less immunogenicity [26], this drug is the best for maintenance medications. Other upcoming new medications will be developed as potential candidates to combine with TAC induction for ASUC treatment in the future. Thus, TAC is a distinct induction therapy for patients with ASUC refractory to corticosteroids.

Our data suggested that intravenous TAC was superior to oral TAC for the treatment of ASUC in short-term efficacy. To our knowledge, no reports describe intravenous TAC for ASUC treatment and our report will be the first and largest one. The limitations of this study include our single-center evaluation of a distinct patient population without oral- intravenous random assignment. Moreover, further prospective studies are needed to confirm our results, with more ASUC patients who failed anti-TNF-α therapies. Answering these questions provides us with further knowledge about the treatment of ASUC.

In summary, TAC is an effective and safe treatment for ASUC refractory to corticosteroid. Intravenous and oral administration of TAC exhibited comparable safety, and it is suggested that intravenous TAC was superior to oral administration in the two-week remission rate and 24-week colectomy-free survival. Intravenous TAC provided not only the fastest achievement of effective concentrations but also an easier and more stable adjustment in the concentration. As TAC is still one of the most promising induction therapies for patients with ASUC, intravenous TAC can be a better option to escape both acute severe disease and emergent colectomy.

## Data Availability

All data analyzed in this study are available from the corresponding author on reasonable request.
